# Supplementary material for: Comprehensive Annotation and Functional Exploration of MicroRNAs in Lettuce
Source: Front Plant Sci. 2021 Dec 24;12:781836. doi: 10.3389/fpls.2021.781836 (PMC8739914; doi:10.3389/fpls.2021.781836)
Supplement: Supplementary file 3 [file Data_Sheet_9.ZIP › GSM2230752.plot/Lsa-miR6118_Lsat_1_v5_gn_1_22801.1_354_TPlot.pdf]

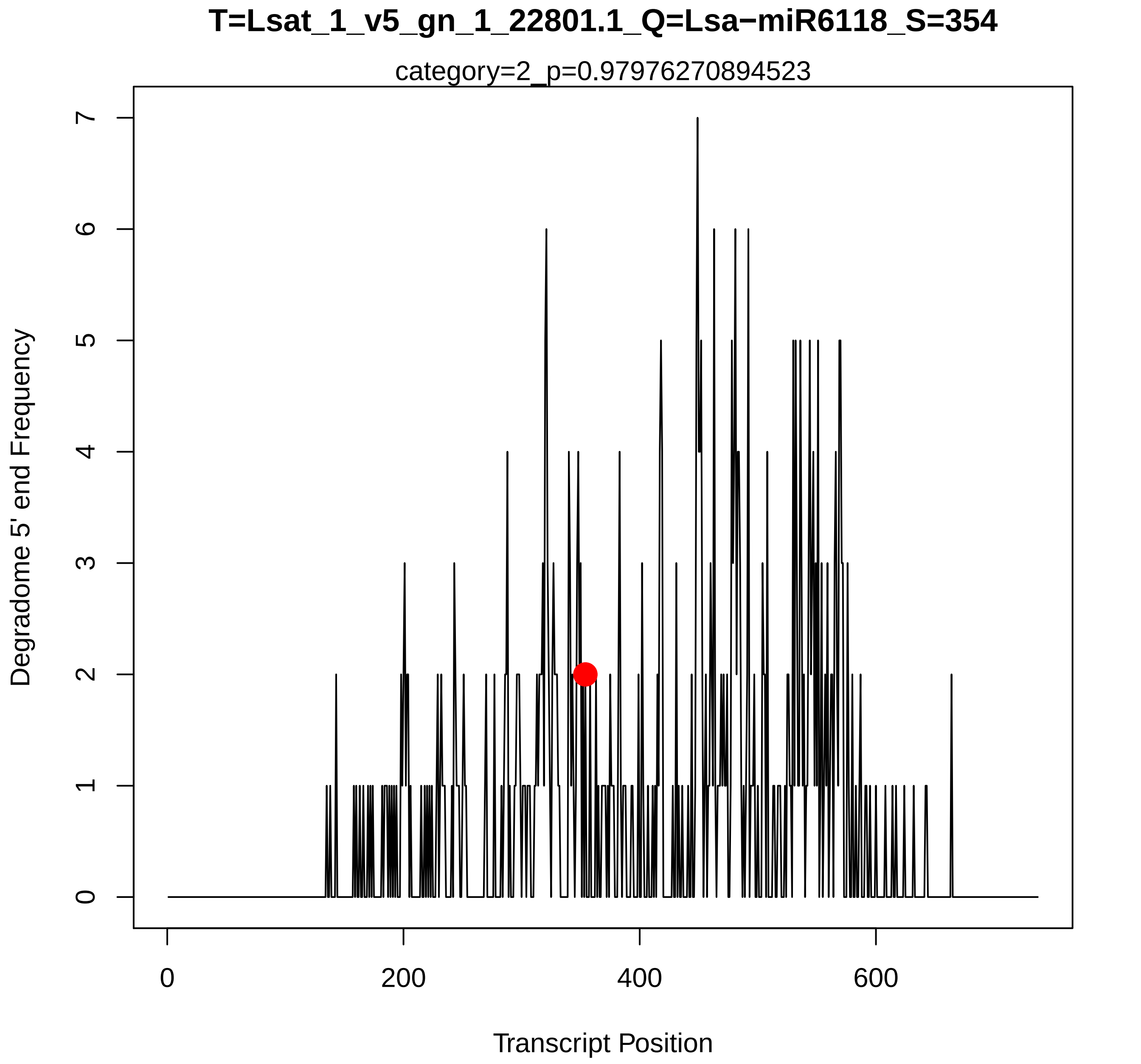

T=Lsat_1_v5_gn_1_22801.1_Q=Lsa−miR6118_S=354
category=2_p=0.97976270894523
Degradome 5' end Frequency
0
1
2
3
4
5
6
7
Transcript Position
0
200
400
600
